# Patterns of survival in patients with recurrent mantle cell lymphoma in the modern era: progressive shortening in response duration and survival after each relapse

**DOI:** 10.1038/s41408-019-0209-5

**Published:** 2019-05-20

**Authors:** Anita Kumar, Fushen Sha, Ahmed Toure, Ahmet Dogan, Andy Ni, Connie L. Batlevi, Maria Lia M. Palomba, Carol Portlock, David J. Straus, Ariela Noy, Steven M. Horwitz, Alison Moskowitz, Paul Hamlin, Craig H. Moskowitz, Matthew J. Matasar, Andrew D. Zelenetz, Anas Younes

**Affiliations:** 1 Lymphoma Service, Memorial Sloan Kettering Cancer Center, New York, NY USA; 2 Department of Pathology, Memorial Sloan Kettering Cancer Center, New York, NY USA; 3 Department of Epidemiology and Biostatistics, Memorial Sloan Kettering Cancer Center, New York, NY USA

**Keywords:** B-cell lymphoma, B-cell lymphoma

## Abstract

As the survival of patients with mantle cell lymphoma (MCL) continues to improve, patients are increasingly being treated with multiple regimens. However, outcome after each line remains poorly characterized in the modern era. To address this knowledge gap, we retrospectively studied 404 consecutive MCL patients who were managed between 2000 and 2014 at Memorial Sloan Kettering Cancer Center. Histologic diagnosis was centrally confirmed, and patients were followed longitudinally from diagnosis throughout their disease course. Progression-free survival (PFS) and overall survival (OS) were determined by Kaplan–Meier method. The median OS and PFS after first-line treatment were 9.7 and 4.0 years, respectively. After second-line therapy, the median OS and PFS were 41.1 and 14.0 months, third line were 25.2 and 6.5 months, and fourth line were 14.4 and 5.0 months. In patients less than 65 years, stem cell transplant (SCT)-based frontline regimens were associated with improved PFS compared with non-SCT regimens (median PFS: 86.2 versus 40.0 months; *P* < 0.01), with a trend toward longer OS (median OS: 165.0 versus 120.0 months; *P* = 0.06). Early treatment failure after first-line regimens was associated with worse OS (5.9 versus 2.5 years; *P* < 0.01). Our study should facilitate establishing proper endpoints for future clinical trials using novel treatment approaches.

## Introduction

Mantle cell lymphoma (MCL) is a rare subtype of B-cell non-Hodgkin lymphoma with distinctive clinical and pathological features. Most patients present with advanced stage disease that is not eradicated by available frontline therapies^1–3^. The treatment of MCL evolved over the past two decades and an associated improvement in survival has been observed. At the present time, chemoimmunotherapy with or without autologous stem cell transplant (ASCT) consolidation is considered the standard of care^4,5^. In some cases, rituximab maintenance after certain regimens has been shown to prolong survival^4,6^. Despite this progress, MCL is considered incurable with current treatment modalities. Disease relapse is almost universal, and most patients require multiple lines of treatment in their lifetime^7–11^.

As the survival of patients with MCL continues to improve, patients are increasingly requiring multiple treatment regimens; however, treatment outcome after each line of therapy remains poorly characterized in the modern era. It is generally asserted in MCL that the remission duration is the longest after frontline therapy and successively shortens with subsequent lines of therapy; however, there is limited published data to characterize patterns of survival in patients with recurrent MCL. Most previously published studies that address treatment outcome in MCL were generated in the pre-rituximab and pre-novel agents era. Furthermore, these studies lacked patient-level longitudinal follow up, or were based on prospective clinical trials using a specified therapy in a heterogeneous patient population with short-term follow up. To address this knowledge gap, we conducted a retrospective chart review of newly diagnosed patients with MCL who were managed at MSKCC and followed their disease course and treatment outcome from 2000 to 2014. This study provides a unique insight on treatment outcome in patients undergoing multiple lines of therapy, which may help guide future drug development for patients with R/R MCL.

## Patients and methods

The study was approved by the institutional review board of MSKCC. Informed consent was obtained from all subjects. We identified 780 consecutive patients with MCL who were evaluated at MSKCC between 2000 and 2014. We excluded 376 patients from this analysis: 283 were seen for a second opinion and lacked a longitudinal follow up at MSKCC, 71 had R/R MCL at initial consult, 19 had no histological confirmation of MCL, and 3 were excluded for other reasons. Thus, 404 patients with histologically confirmed MCL who were initially managed and subsequently followed at MSKCC were included in this analysis. All pathology was centrally reviewed by MSKCC and met criteria for MCL as defined by the World Health Organization classification system^12^. Initial observation after tumor diagnosis was defined as treatment deferral for at least 3 months after diagnosis with documentation of an intent to observe by the primary oncologist.

Response assessment to therapy was based on the treating physician's assessment, incorporating data from imaging studies, and when appropriate, a tissue biopsy. Early treatment failure of first-line regimens was defined as failure to achieve a complete response (CR) at the completion of first-line therapy, or disease relapse requiring second-line therapy within 12 months of receiving frontline stem cell transplant (SCT) or non-transplant regimen. Late treatment failure of first-line therapy was defined as progression of disease after achieving a CR and did not meet the criteria of early treatment failure.

MCL international prognostic index (MIPI) was calculated using age, lactate dehydrogenase (LDH) level, Eastern Cooperative Oncology Group (ECOG) performance status and WBC count as [0.03535 × age (years)] + 0.6978 (if ECOG > 1) + [1.367 × log10 (LDH/ULN)] + [0.9393 × log10 (WBC counts per µL)]. Cutoff values of 5.7 and 6.2 were used to define low-, intermediate-, and high-risk groups^13^. Objective response rate is defined as the CR rate plus partial response (PR) rate. Progression-free survival (PFS) is defined as the time from the initiation of the therapy until the date of disease progression or death from any cause. Disease relapse or progression was defined as appearance of new symptoms or signs of the disease that was confirmed pathologically or radiographically. Overall survival (OS) is defined as the time from the date of initiation of therapy until the date of death from any cause. Survival was estimated by the Kaplan–Meier method, and comparisons were made by log-rank tests. Comparisons of OS and PFS of multiple lines of therapy were made by Cox regression method with robust variance estimation to account for within-patient correlation among the outcomes. Hazard ratio (HR) was calculated using Cox regression method. Patients who were lost to follow-up were censored from the date and status when last known to be alive. Statistical significance was defined as *P* < 0.05 and all statistical analyses were completed with R 3.5.0

## Results

### Patient characteristics

**Fig. 1:**
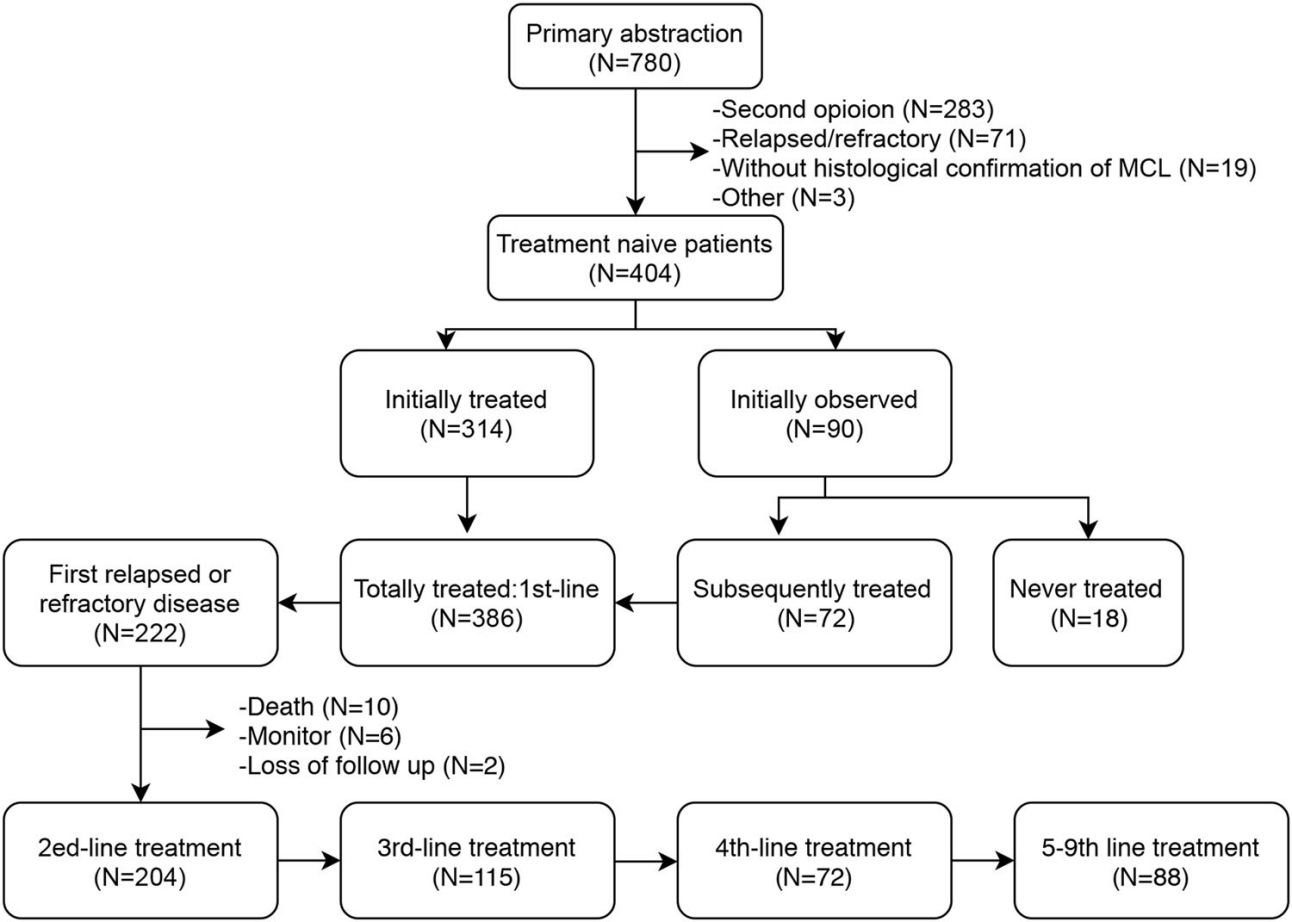
Patient inclusion and treatment overview

From Feb. 2000 to Dec. 2014, a total of 404 patients with a new diagnosis of MCL initially managed at MSKCC were identified, 22% (90 of 404) were initially observed, of whom 20% (18 of 90) never required therapy, and 80% (72 of 90) subsequently required therapy (Fig. 1). A total of 386 patients received first-line treatment, of whom 222 (58%) patients had relapsed or refractory disease. For the documented 204 second-line treatments, 203 were for disease relapse or progression (one patient changed therapy due to liver toxicity after one cycle of first-line chemotherapy without evidence of disease progression at the initiation of second-line treatment). Patients’ characteristics are shown in Table 1.

**Table 1:** Baseline demographic at initial diagnosis and at first disease relapse or progression

	At diagnosis (*N* = 404)	At first relapse or progression (*N* = 222)
Characteristics	Number (%)	Number (%)
Age: median (range)	64 (28–90)	69 (37–91)
≥65	189 (47%)	145 (65%)
Sex		
Female	98 (24%)	45 (20%)
Male	306 (76%)	177 (80%)
Stage at initial diagnosis		–
Incomplete staging	13 (3%)	–
I	17 (4%)	–
II	15 (4%)	–
III	26 (6%)	–
IV	333 (82%)	–
Status after first-line treatment	–	
Early failure	–	70 (32%)
Late failure	–	152 (68%)
ECOG		
0	256 (64%)	78 (37%)
1	109 (27%)	103 (49%)
≥2	33 (8%)	31 (15%)
Missing	6	10
LDH/ULN ratio		
≥1	130 (37%)	64 (33%)
<1	219 (63%)	129 (67%)
Missing	55	29
WBC, ×10^9^/L		
Missing	37	20
Assessable	367	202
median (range)	7.2 (2.5–483.6)	6.2 (2.0–208.5)
MIPI score		
Median (range)	5.89 (3.59–8.85)	5.91 (3.25–8.42)
Low risk	124 (36%)	66 (35%)
Intermediate risk	113 (33%)	56 (30%)
High risk	110 (32%)	67 (35%)
Missing	57	33
Ki-67^a^		
<10%	26 (11%)	5 (5%)
10% to <30%	99 (43%)	26 (27%)
≥30%	105 (46%)	64 (67%)
Missing	174	127
Histology		
Blastoid/pleomorphic	32 (8%)	39 (27%)
Nonblastoid/pleomorphic	372 (92%)	108 (73%)
Missing	0	75

*ECOG* eastern cooperative oncology group, *LDH* lactate dehydrogenase, *MIPI* mantle cell lymphoma international prognostic index *UNL* upper limit of normal

^a^Ki-67 of the tissue samples except for bone marrow

### Treatments

Three hundred and seven (96%) of the patients were treated with first-line systemic therapy, which included anti-CD20 antibody in 350 (95%) of the patients. Chemotherapy induction followed by SCT was offered to 46% (*n* = 179) of patients. Nontransplant regimens included bendamustine-based regimens (*n* = 54, 14%) and R-CHOP (rituximab, cyclophosphamide, doxorubicin, vincristine, and prednisone)-based regimen (*n* = 42, 11%). Other regimens are summarized in Supplementary Table 1. Two hundred and four patients received second-line treatment, which included SCT in 26 (13%, 16 allogeneic SCT and 10 ASCT) (Table 2). Most common nontransplant based second-line treatments were ibrutinib-based (23%, *n* = 46), bortezomib-based (15%, *n* = 30), and bendamustine-based regimens (15%, *n* = 30). A number of investigational agents were used in the clinical trial setting.

**Table 2:** Regimen used as second-line treatment (*N* = 204)

Content	No. (%)
Induction followed by SCT	26 (12.7%)
AlloSCT	16 (7.8%)
AutoSCT	10 (4.9%)
Without SCT consolidation	178 (87.3%)
Ibrutinib-based regimen	46 (22.5%)
Ibrutinib single agent	29 (14.2%)
Ibrutinib plus chemotherapy/rituximab	9 (4.4%)
Ibrutinib plus buparlisib	8 (3.9%)
Bortezomib-based regimens	30 (14.7%)
Bortezomib single agent	14 (6.9%)
Bortezomib plus rituximab	5 (2.5%)
Rituximab, cyclophosphamide, bortezomib, and prednisone (R-CBorP)	8 (3.9%)
Bortezomib plus other chemotherapeutic agents	3 (1.5%)
Bendamustine-based regimens	30 (14.7%)
Rituximab plus bendamustine (RB)	22 (10.8%)
Bendamustine plus bortezomib plus rituximab (BBR)	5 (2.5%)
Ofatumumab plus bendamustine	1 (0.5%)
Veliparib plus bendamustine	1 (0.5%)
Bendamustine single agent	1 (0.5%)
Monoclonal antibody single agent	21 (10.3%)
CD20 antibody single agent	18 (8.8%)
CD19 antibody single agent	2 (1.0%)
ADCT-401 single agent	1 (0.5%)
Radiotherapy/surgical resection	17 (8.3%)
Radiotherapy alone	13 (6.4%)
Surgical resection alone	4 (2.0%)
Lenalidomide plus rituximab	5 (2.5%)
PI3K inhibitor single agent	2 (1.0%)
Duvelisib single agent	1 (0.5%)
Buparlisib single agent	1 (0.5%)
BCL2 inhibitor-based regimens	2 (1.0%)
Venetoclax single agent	1 (0.5%)
BCL201 plus idelalisib	1 (0.5%)
CDK 4/6 inhibitor palbociclib single agent	1 (0.5%)
HDAC inhibitor vorinostat single agent	1 (0.5%)
SYK/JAK inhibitor cerdulatinib single agent	1 (0.5%)
Other	22 (10.8%)
Other conventional chemotherapies*	21 (10.3%)
Radioimmunotherapy	1 (0.5%)

*SCT* stem cell transplant, *alloSCT* allogenic SCT, *AutoSCT* autologous SCT

Asterisk indicates other conventional chemotherapy regimens used including PC (pentostatin,cyclophosphamide) ± rituximab, *N* = 5; R-CHOP (rituximab, cyclophosphamide, doxorubicin, vincristine, and prednisone), *N* = 4; EPOCH (etoposide, prednisone, vincristine, cyclophosphamide, and doxorubicin) ± rituximab, *N* = 2; ICE (ifosfamide, carboplatin, and etoposide) ± rituximab, *N* = 2; FC (fludarabine and cyclophosphamide) ± rituximab, *N* = 2; CEPP (cyclophosphamide, etoposide, procarbazine, and prednisone), *N* = 1; FM (fludarabine and mitoxantrone), *N* = 1; rituximab and methotrexate, *N* = 1; R-BAC (rituximab, bendamustine, and cytarabine), *N* = 1; rituximab and ifosfamide, *N* = 1; R-GCVP(gemcitabine, cyclophosphamide, vincristine, and prednisolone), *N* = 1

### Treatment outcome and survival after first and second-line regimens

**Fig. 2:**
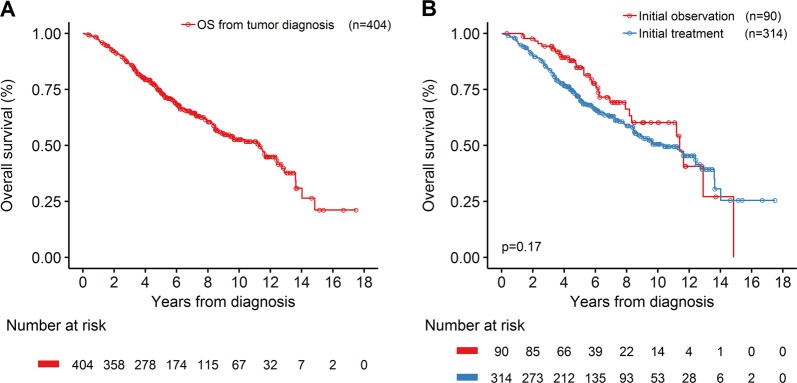
Kaplan–Meier plots of overall survival (OS) since tumor diagnosis. **a** OS for 404 patients since tumor diagnosis. **b** OS by initial observation or initial treatment after tumor diagnosis (*p* = 0.17)

**Fig. 3:**
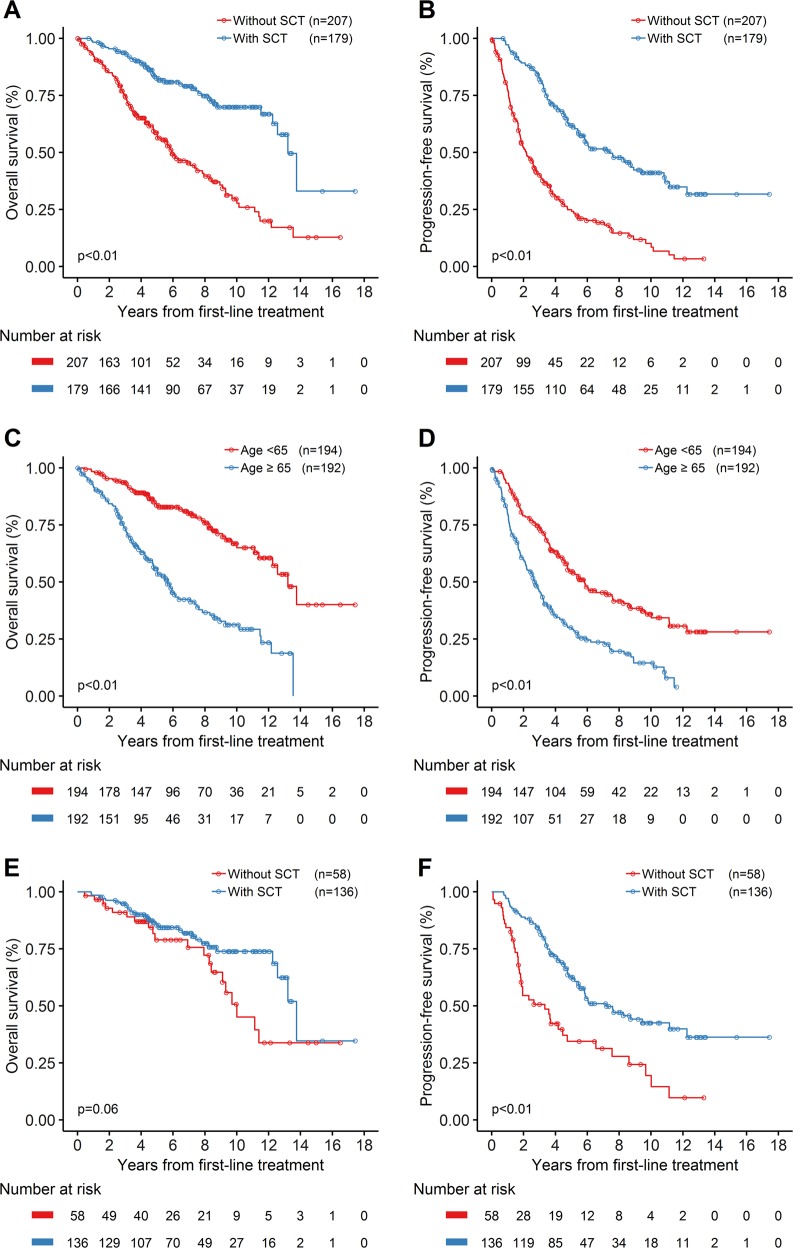
Kaplan–Meier plots of overall survival (OS) and progression-free survival (PFS) in patients with mantle cell lymphoma treated with first-line therapy. **a**, **b** OS and PFS for patients with or without upfront stem cell transplantation (SCT) as consolidation. **c**, **d** OS and PFS for patients older or younger than 65 years when first-line treatment was commenced. Patients older than 65 years had an inferior median OS (67.6 months; 95% CI, 57.1–85.0 months versus 158.5 months; 95% CI, 136.6–NR months; *P* < 0.01) and median PFS (32.3 months; 95% CI, 25.5–38.3 months versus 69.8 months; 95% CI, 56.8–91.5 months; *P* < 0.01). **e**, **f** OS and PFS for patients younger than 65 years when first-line treatment was commenced, with or without upfront SCT as consolidation. SCT was associated with a statistically significant difference in PFS (median PFS: 86.2 months; 95% CI, 65.4–147.0 months versus 40.0 months; 95% CI, 21.6–56.8 months; *P* < 0.01), and a trend towards improvement in OS (median OS: 165.0 months; 95% CI, 151.0–NR months versus 120.0 months; 95% CI, 101.0–NR months; *P* = 0.06)

With a median follow-up for surviving patients of 74.0 months (range: 4.1–209.9 months), the median OS for the entire group (*n* = 404) was 11.25 years (135 months; 95% CI, 104.0–149.0 months; Fig. 2a). There was no significant difference in survival between patients who were initially observed versus immediately treated (median OS: 137.0 months; 95% CI, 98.1–not reached (NR) months; versus 125.0 months; 95% CI, 101.0–152.0 months; *P* = 0.17; Fig. 2b). Patients who received upfront SCT had significantly better median OS (158.5 months; 95% CI, 147.0–NR months; versus 71.1 months; 95% CI, 60.2–94.1 months; *P* < 0.01; Fig. 3a) and median PFS (88.7 months; 95% CI, 65.8–113.4 months; versus 25.9 months; 95% CI, 21.3–32.3 months; *P* < 0.01; Fig. 3b). Patients older than 65 years had an inferior median OS (*P* < 0.01; Fig. 3c) and median PFS (*P* < 0.01; Fig. 3d). However, when the analysis was restricted to patients who are younger than 65 years of age, SCT consolidation as part of first-line regimens provided a statistically significant difference in PFS (*P* < 0.01; Fig. 3f), and a trend towards improvement in OS (*P* = 0.06; Fig. 3e).

**Fig. 4:**
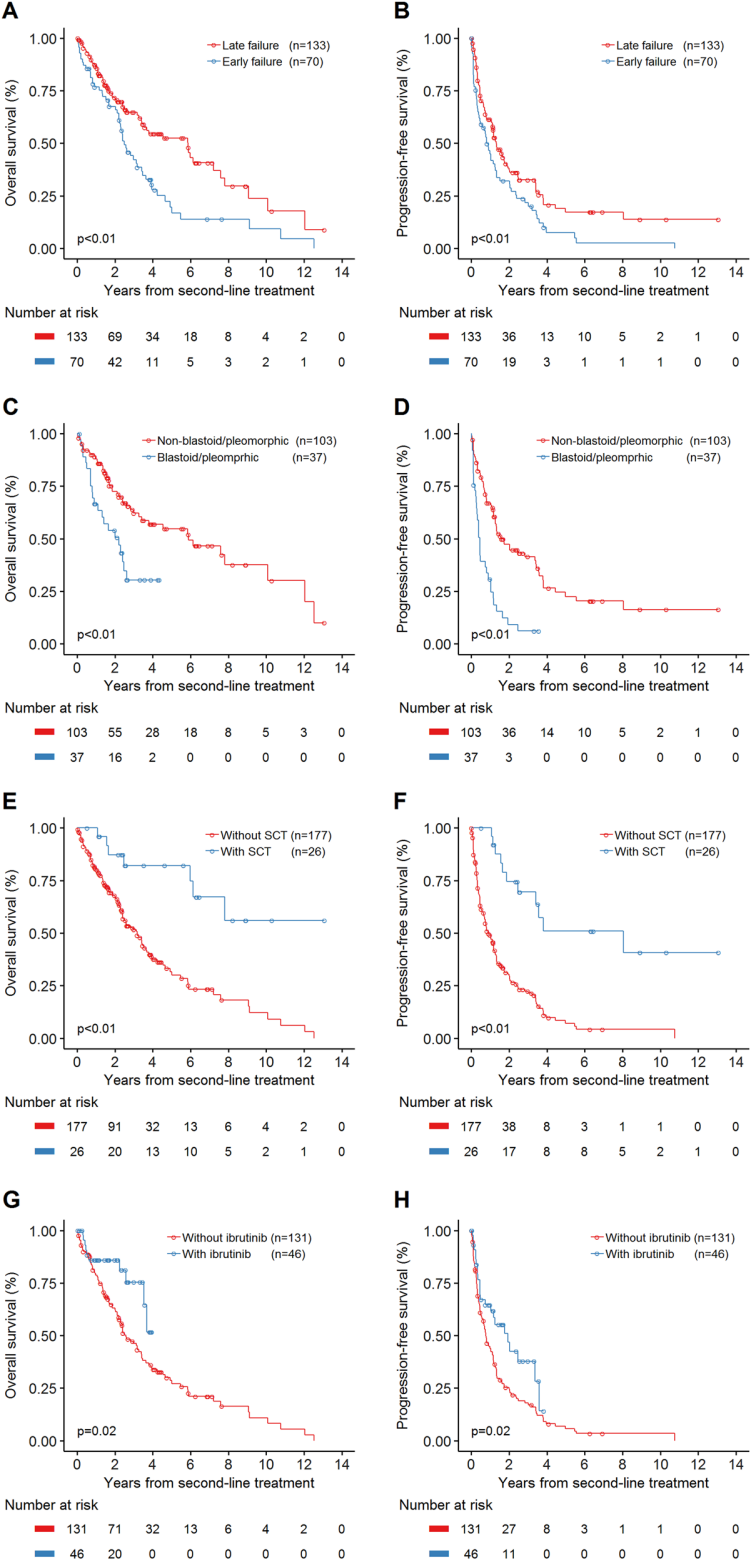
Kaplan–Meier plots of overall survival (OS) and progression-free survival (PFS) in patients with mantle cell lymphoma treated with second-line therapy. **a**, **b** OS and PFS for patients with early or late failure after first-line treatment. Patients who failed later to first-line treatment had superior median OS (70.4 months; 95% CI, 41.0–86.4 months versus 29.9 months; 95% CI, 26.4–41.1 months; *P* < 0.01) and median PFS (15.7 months; 95% CI, 13.4–21.0 months versus 9.7 months; 95% CI, 4.9–15.2 months; *P* < 0.01) when compared with patients who failed early. **c**, **d** OS and PFS for patients with or without blastoid/pleomorphic histology. Patients with blastoid or pleomorphic histology had inferior median OS (26.5 months; 95% CI, 10.9–31.4 months versus 70.8 months; 95% CI, 39.2–120.9 months; *P* < 0.01) and median PFS (5.4 months; 95% CI, 3.0–9.7 months versus 18.7 months; 95% CI, 15.1–40.9 months; *P* < 0.01). **e**, **f** OS and PFS for patients with or without stem cell transplantation (SCT) at second line. Patients with SCT consolidation in second remission had significantly better median OS (NR; 95% CI, 71.8-NR months versus 38.0 months; 95% CI, 28.8–44.2 months; *P* < 0.01) and median PFS (96.4 months; 95% CI, 29.6-NR months versus 10.9 months; 95% CI, 8.1–14.1 months; *P* < 0.01). **g**, **h** Among patients without SCT at second line, OS, and PFS by second-line treatment with or without ibrutinib. The addition of ibrutinib into salvage regimen was associated with improved median OS (NR; 95% CI, 42.4-NR months versus 31.1 months; 95% CI, 26.4–41.0 months; *P* = 0.02) and median PFS (23.3 months; 95% CI, 8.8–40.4 months versus 9.1 months; 95% CI, 6.6–13.4 months; *P* = 0.02)

Patients who had a late treatment failure after first-line treatment had a superior outcome when compared with patients who had an early treatment failure (Fig. 4a, b). Patients with blastoid or pleomorphic histology had inferior treatment outcome (Fig. 4c, d). Twenty-six patients received second-line treatment followed by SCT (16 allogeneic and 10 autologous), and those patients had a better median OS and PFS when compared with those who did not receive SCT (Fig. 4e, f). Similarly, patients who received ibrutinib as part of their salvage therapy had improvement in their median OS and PFS (Fig. 4g, h). Patients younger than 65 years at the time of initiating second-line treatment had longer survival (median PFS and median OS: 16.3 and 93.6 months, respectively) compared with those older than 65 years (median PFS and median OS: 12.3 and 34.0 months, respectively, Supplementary Fig. 1A, B). The MIPI index is a well-established prognostic factor in patients with newly diagnosed MCL^13^. Our data showed secondary MIPI also had a prognostic value at the time of initiating second-line therapy (Supplementary Fig. 1C, D**)**.

### Outcomes after multiple lines of therapy

**Fig. 5:**
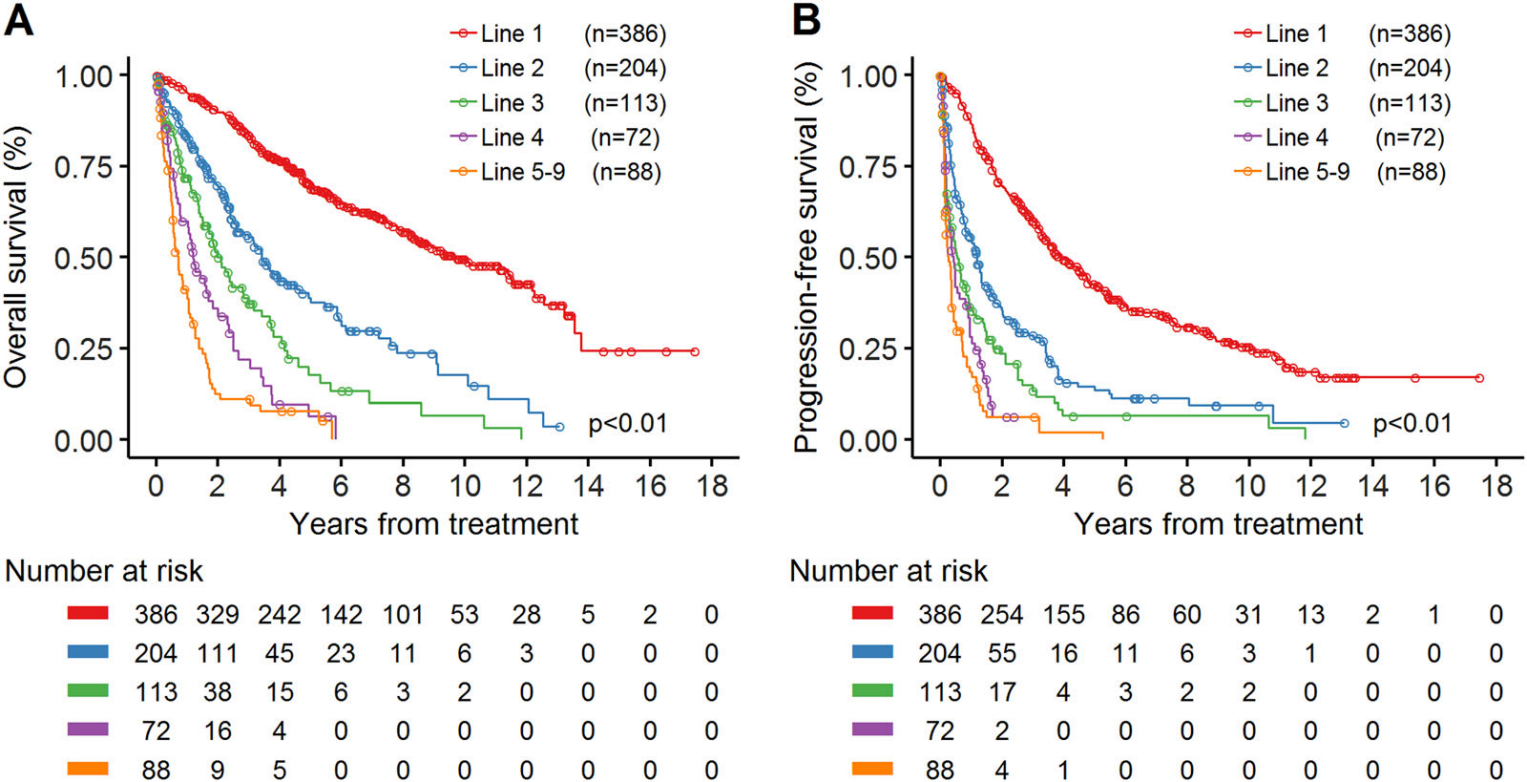
Kaplan–Meier plots of overall survival (OS) and progression-free survival (PFS) in patients with mantle cell lymphoma after multiple lines of therapy. **a**, **b** OS and PFS after treatment with line 1, line 2, line 3, line 4, and line 5–9

Treatment outcome declined with successive lines of treatment. As the line of treatment increased, the percentage of patients achieving CR decreased (Supplementary Fig. 2). We also calculated the OS and PFS following each line of therapy. The median OS following lines 1, 2, 3, 4, and 5–9 were 116.3 months (95% CI, 99.1–145.9 months); 41.1 months (95% CI, 31.1–54.5 months); 25.2 months (95% CI, 17.9–33.8 months); 14.4 months (95% CI, 9.2–22.0 months) and 8.6 months (95% CI, 6.4–12.1 months), respectively (Fig. 5a, Supplementary Table 2). The median PFS following lines 1, 2, 3, 4, and 5–9 were 47.4 months (95% CI, 40.5–56.5 months); 14.0 months (95% CI, 9.7–16.0 months); 6.5 months (95% CI, 3.8–10.0 months); 5.0 months (95% CI, 3.0–9.7 months), and 3.2 months (95% CI, 2.0–4.2 months), respectively (Fig. 5b, Supplementary Table 2).

A total of 77 patients with R/R MCL received ibrutinib-based therapy between 2012 and 2018, with a median prior lines of therapy of 1 (range 1–7). Patients who received ibrutinib-based therapy at second line had a better survival compared with patients who received ibrutinib-based therapy at third line or beyond. (OS: HR, 0.27, *P* < 0.01; PFS: HR, 0.52; *P* = 0.03, Supplementary Fig. 3).

## Discussion

A unique aspect of our study is reporting patient-level longitudinal data of treatment outcome from the time of initial diagnosis throughout the course of the disease. We found that the median OS of MCL patients who are diagnosed after 2000 to be 11.25 years. This represents remarkable progress, as the OS of patients treated before the 1990s was reported to be less than 3 years^14^. The improvement in OS is likely due to improved outcome of frontline regimens, and the introduction of several new agents.

As the survival of MCL patients continues to improve, patients are increasingly requiring additional lines of therapy. Thus, future drug development may not only aim at improving treatment outcome of second-line therapy, but also will aim at introducing new agents that could be approved by regulatory agencies after failing second, third, or fourth line of therapy. Thus, it is important to benchmark treatment outcome by line of therapy and describe the natural history of the disease over time and after multiple recurrences. In our study, we observed a progressive shortening in the response duration and survival after successive lines of therapy. The median PFS after second, third, fourth, and ≥5th lines of therapy were 14, 6.5, 5, and 3.3 months, respectively. Rule et al.^15^ reported a similar observation of shortened PFS by line of therapy when patients were treated with ibrutinib. Accordingly, our data indicate that MCL patients failing at least two lines of therapy are a high-risk group with a shortened median time to subsequent relapse and death. New agents that are tested in this patient population should aim at achieving a PFS that exceeds 6.5 months to be clinically meaningful.

A previous study from the United Kingdom Haematological Malignancy Research Network (HMRN) reported a median survival after second-line therapy of 0.8 years and OS of 0.6, 0.4, and 0.1 years after third-, fourth-, and fifth-line of treatment, respectively. The inferior OS reported in the British study compared to the current study is likely due to multiple factors, including differences in the median age, variable administration of anti-CD20 antibody, limited use of SCT consolidation, and availability of active investigational agents^16^.

A previous study has shown that patients with MCL who relapsed within 12 months after ASCT had a dismal prognosis with a median OS of 6 months^17^. Our study used a similar definition for the early treatment failure, which included patients who initiated second-line treatment within 12 months after upfront SCT. Our study confirms that patients who have early treatment failure after first-line therapy have a poor prognosis. In our study, the median PFS and OS for these patients were 9.6 and 29.9 months, respectively.

In this report, patients younger than 65 years who received consolidation SCT in the first remission had a better PFS compared with those who did not, and a trend towards improved OS, but this difference didn’t meet statistical significance (*P* = 0.06). The lack of OS advantage for those who received SCT as part of their first-line therapy may be due to patient selection and the limitations of a retrospective analysis. Alternatively, this may be due to the fact that SCT in the second-line setting was also beneficial for younger patients with favorable baseline features, as our data indicated. Ongoing randomized studies, such as TRIANGLE trial (NCT02858258) and NCTN Trial (NCT03267433) will adequately address the role of SCT in first-line regimens. However, a retrospective study from the Center for International Blood and Marrow Transplant Research demonstrated that ASCT may also offer survival benefit later in the MCL disease course (beyond first CR)^18^. P53 mutation has been shown to be a prognostic factor in MCL, especially in transplant eligible patients^19^. The P53 mutation status for all patients, however, are not comprehensively assessed in our retrospective database, making robust comparative statistical analysis less feasible.

Although ibrutinib was the most commonly used drug in second-line treatment in our patients, it only accounted for 22.5% of second-line therapy, in part reflecting the fact that ibrutinib was FDA-approved in 2013 and was not available for all patients at the time of relapse outside of clinical trials. The relatively small proportion of patients received ibrutinib-based regimen as second-line treatment is one of the limitations of this analysis. Nevertheless, the results of this study confirm the data published by Rule et al. that the efficacy of ibrutinib is greatest when utilized in earlier line of therapy^15^. In addition, the wide spectrum of therapeutic approaches in our study suggests that there is no consensus in clinical practice regarding the optimal therapy for R/R MCL, even within a single institute. Despite the availability of multiple FDA-approved novel agents with distinctive molecular targets and encouraging overall response rates, the majority of responses are partial, and the durability of response is often short. Our study demonstrated that there remains a clinical need for novel therapeutic approaches particularly in patients with early failure or are multiple relapsed. Our analysis of treatment outcome after each line of therapy provides a new benchmark in the modern era that could guide future drug development in MCL.

## Supplementary information

Supplemental material
