# Supplementary material for: Patterns of survival in patients with recurrent mantle cell lymphoma in the modern era: progressive shortening in response duration and survival after each relapse
Source: Blood Cancer J. 2019 May 20;9(6):50. doi: 10.1038/s41408-019-0209-5 (PMC6527702; doi:10.1038/s41408-019-0209-5)

# **Supplemental data**

Patterns of survival in patients with recurrent mantle cell lymphoma in the modern era: progressive shortening in response duration and survival after each relapse

Anita Kumar^1*^, Fushen Sha^1*^, Ahmed Toure^1^, Ahmet Dogan^2^, Andy Ni^3^, Connie L. Batlevi^1^, Maria Lia M. Palomba^1^, Carol Portlock^1^, David J. Straus^1^, Ariela Noy^1^, Steven M. Horwitz^1^ Alison Moskowitz^1^, Paul Hamlin^1^, Craig H. Moskowitz^1^, Matthew J. Matasar^1^, Andrew D. Zelenetz^1^, Anas Younes^1^

1: Lymphoma Service, Memorial Sloan Kettering Cancer Center, New York, NY

2: Department of Pathology, Memorial Sloan Kettering Cancer Center, New York, NY

3: Department of Epidemiology and Biostatistics, Memorial Sloan Kettering Cancer Center, New York, NY

* Contributed equally to this work

**Corresponding Author:**

Anita Kumar

Memorial Sloan Kettering Cancer Center

Box 468, 1275 York Av

New York, NY, 10065

Phone: 212-639-3127

Fax: 212-717-3036

Email: [kumara2@mskcc.org](mailto:kumara2@mskcc.org)

**Running title:** Outcomes of R/R MCL

**Word count**: *Blood Cancer journal*

Article type: Article

Title characters: 163

Running title characters: 19

Abstract: 200 (limit: 200 words)

Text: 2291 (limit: 4500 words)

Figure/Tables: 2 tables, 5 figures, 2 supplemental tables and 3 supplemental tables. (limit: 8 total figures and tables).

Reference: 19 (limit: 60)

Keywords: mantle cell lymphoma, outcome, multiple lines

**Supplemental Table 1**

| **Supplemental Table1.** First-line treatment (N=386) | |
| --- | --- |
| Content | No. (%) |
| Induction followed by SCT | 179 (46.4%) |
| autoSCT | 172 (44.6%) |
| alloSCT | 7 (1.8%) |
| Without upfront SCT | 207 (53.6%) |
| Bendamustine-based regimen | 54 (14.0%) |
| R-bendamustine | 35 (9.1%) |
| Ofatumumab plus bendamustine | 19 (4.9%) |
| R-CHOP-based regimen | 42 (10.9%) |
| Radioimmunotherapy | 25 (6.5%) |
| Intensive chemotherapy | 24 (6.2%) |
| R-CHOP+R-ICE | 14 (3.6%) |
| Cytarabine containing regimen | 10 (2.6%) |
| Anti-CD20 antibody monotherapy | 17 (4.4%) |
| Rituximab monotherapy | 15 (3.9%) |
| Ofatumumab monotherapy | 2 (0.5%) |
| Radiotherapy alone | 16 (4.1%) |
| Other | 29 (7.5%) |
| Fludarabine-based regimen | 14 (3.6%) |
| R-Bendamustine +/- ibrutinib | 5 (1.3%) |
| Bortezomib monotherapy | 3 (0.8%) |
| Pentostatin-based regimen | 3 (0.8%) |
| Chlorambucil-based regimen | 2 (0.5%) |
| Cladribine monotherapy | 2 (0.5%) |
| alloSCT, allogenic SCT; autoSCT, autologous SCT; R-bendamustine, rituximab and bendamustine;R-CHOP, rituximab, cyclophosphamide, doxorubicin, vincristine, and prednisone; R-ICE, rituximab, ifosfamide, carboplatin and etoposide; SCT, stem cell transplant. | |

**Supplemental Table 2**

| **Supplemental Table 2.** Overall survival and progression-free survival by line of treatment | | | | | | | | |
| --- | --- | --- | --- | --- | --- | --- | --- | --- |
| Line of  treatment | Median OS, months  (95% CI) | 1-y OS rate, %  (95% CI) | 5-y OS rate, %  (95% CI) | 10-y OS rate, %  (95% CI) | Median PFS, months  (95% CI) | 1-y PFS rate, %  (95% CI) | 5-y PFS rate, %  (95% CI) | 10-y PFS rate, %  (95% CI) |
| 1 (n=386) | 116.3 (99.1-145.9) | 95.0 (92.3-96.8) | 69.7 (64.4-74.3) | 49.7 (42.9-56.1) | 47.4 (40.5-56.5) | 86.6 (82.8-89.7) | 42.7 (37.4-47.9) | 25.6 (20.3-31.2) |
| 2 (n=204) | 41.1 (31.1-54.5) | 83.2 (77.2-87.8) | 38.9 (30.5-47.3) | 17.8 (9.1-28.7) | 14.0 (9.7-16.0) | 55.5 (48.3-62.2) | 13.5 (8.2-20.1) | 9.5 (4.7-16.3) |
| 3 (n=113) | 25.2 (17.9-33.8) | 71.9 (62.3-79.5) | 17.8 (9.3-28.6) | 6.7 (1.5-17.6) | 6.5 (3.8-10.0) | 35.4 (26.3-44.6) | 6.7 (2.3-14.3) | 6.7 (2.3-14.3) |
| 4 (n=72) | 14.4 (9.2-22.0) | 59.9 (46.9-70.7) | 6.5 (1.4-17.3) | NA | 5.0 (3.0-9.7) | 28.1 (17.5-39.7) | NA | NA |
| 5-9 (n=88) | 8.6 (6.4-12.1) | 40.0 (29.3-50.5) | 7.9 (3.1-15.8) | NA | 3.2 (2.0-4.2) | 18.5 (10.7-28.0) | NA | NA |
| Abbreviations: CI, confidence interval; OS, overall survival; PFS, progression-free survival. | | | | | | | | |

**Supplemental Figure Legends**

**Supplemental Fig 1**. Kaplan-Meier plots of overall survival (OS) and progression-free survival (PFS) in patients with mantle cell lymphoma treated with second-line therapy. (A-B) OS and PFS for patients older or younger than 65 years when initiating therapy. (C-D) OS and PFS for patients with high-risk (HR), intermediate-risk (IR) or low-risk (LR) secondary MIPI.

**Supplemental Fig 2** Response by lines of treatment.

**Supplemental Fig 3** Kaplan-Meier plots of overall survival (OS) and progression-free survival (PFS) in patients with mantle cell lymphoma treated with ibrutinib-based regimen without SCT consolidation, OS and PFS after line 2 versus line 3 and beyond.

**Supplemental Fig 1**


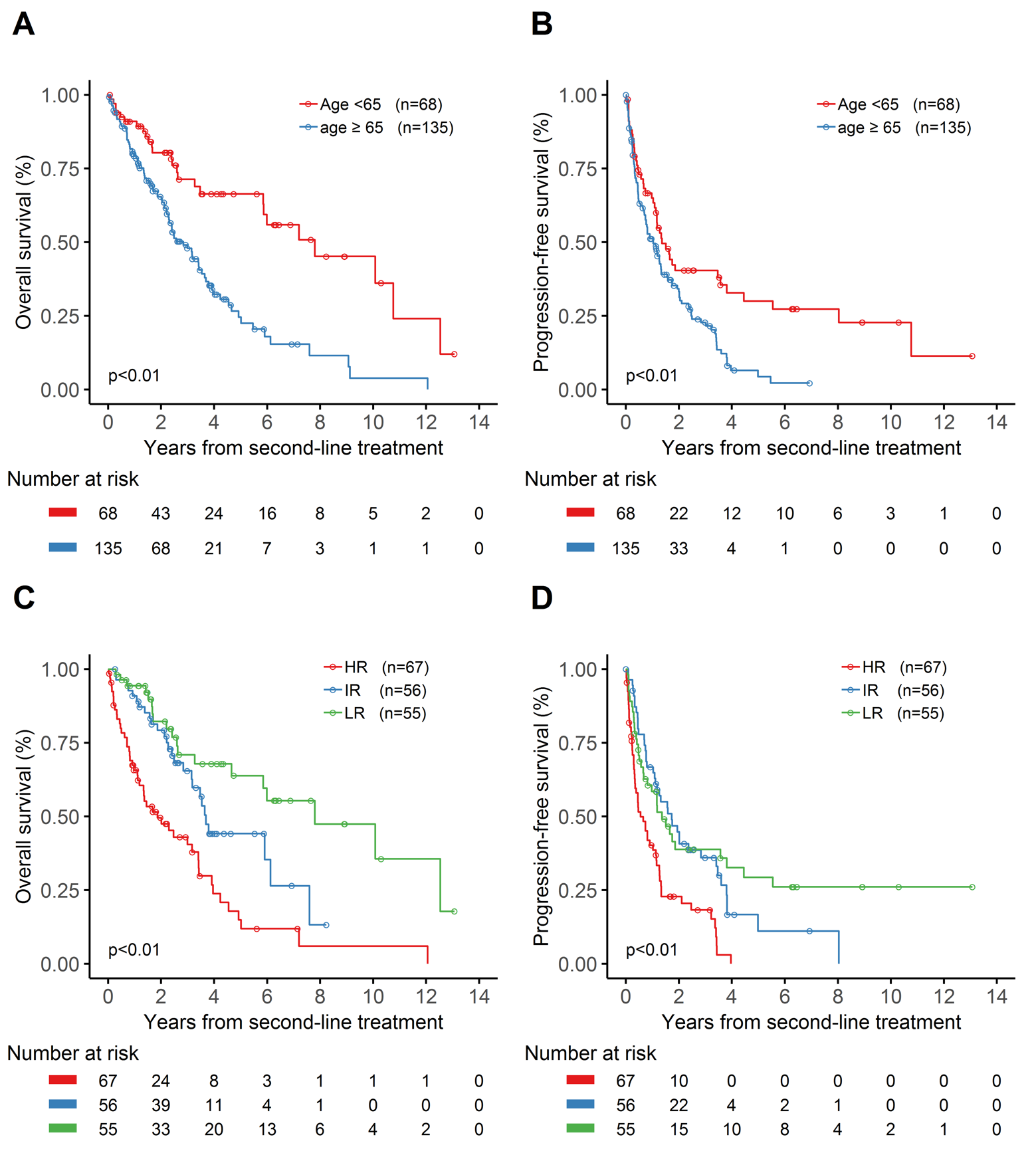


**Supplemental Fig 2**

**
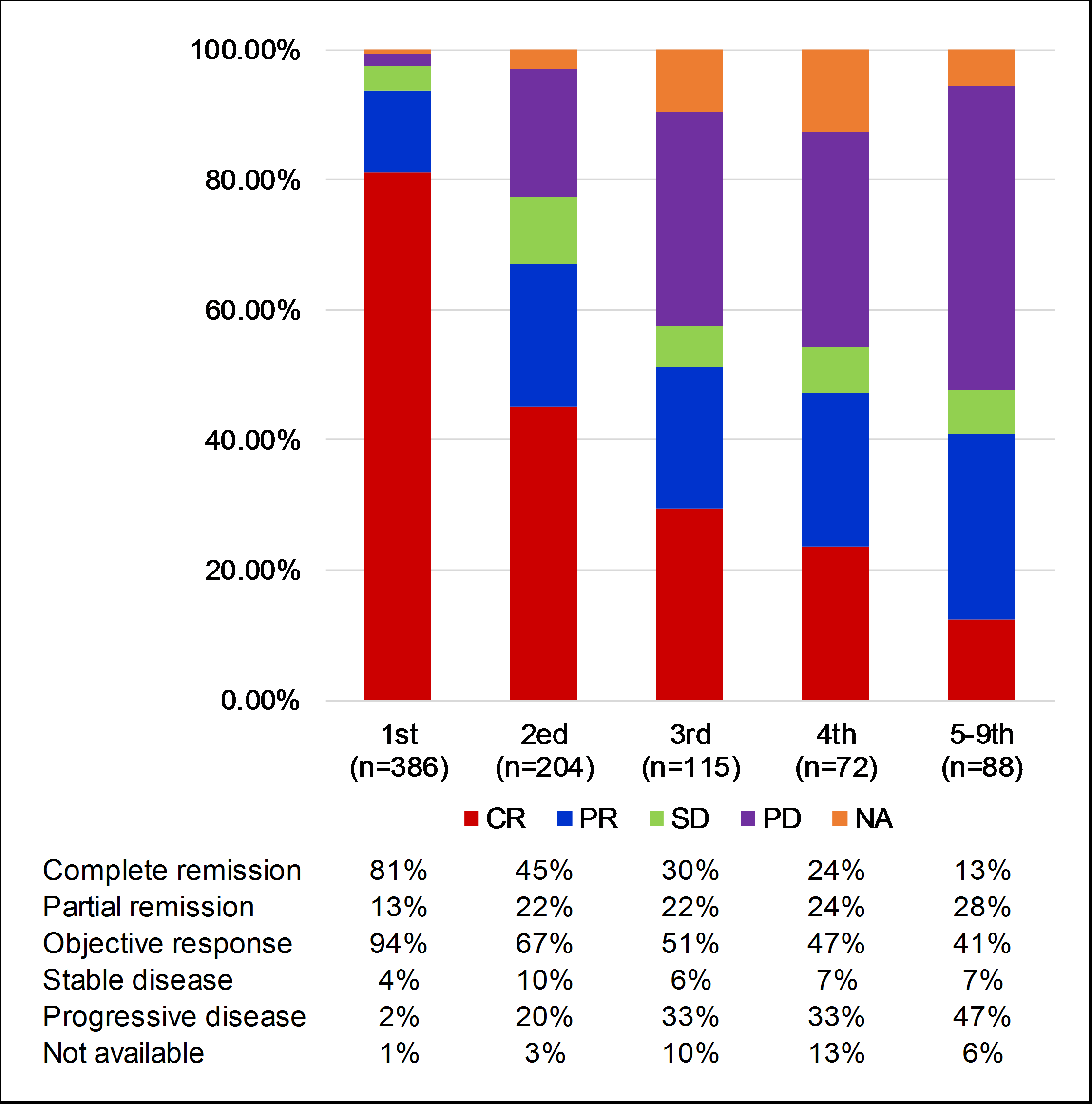
**

**Supplemental Fig 3**


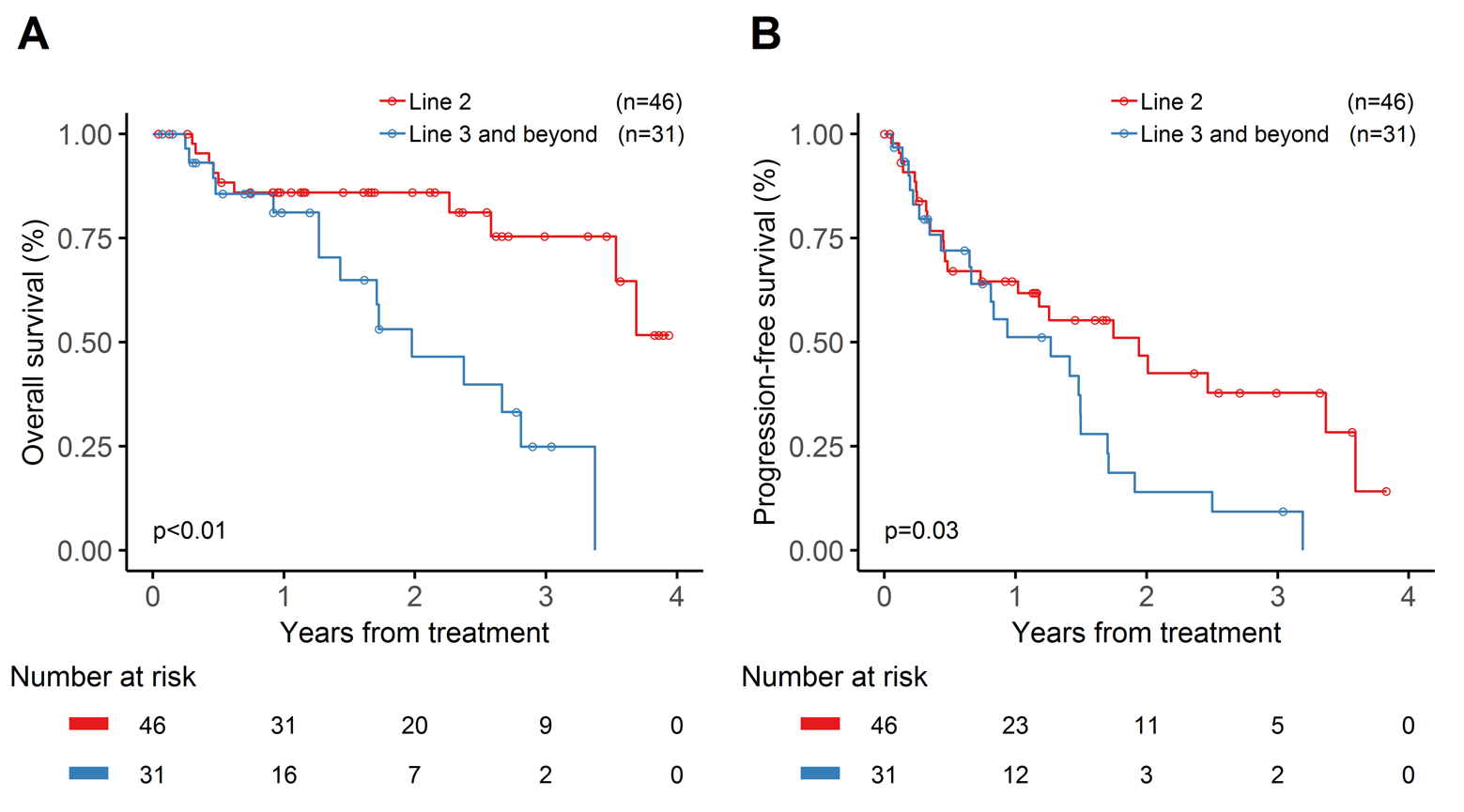

Supplement: Supplementary file 1 — Supplemental material [file 41408_2019_209_MOESM1_ESM.docx]
